# Low dose anti-thymocyte globulin with low dose posttransplant cyclophosphamide (low dose ATG/PTCy) can reduce the risk of graft-versus-host disease as compared with standard-dose anti-thymocyte globulin in haploidentical peripheral hematopoietic stem cell transplantation combined with unrelated cord blood

**DOI:** 10.1038/s41409-020-01047-2

**Published:** 2020-09-01

**Authors:** Xiaoqian Xu, Jun Yang, Yu Cai, Su Li, Jiahua Niu, Kun Zhou, Ying Jiang, Xiaowei Xu, Chang Shen, Chongmei Huang, Huiying Qiu, Daolin Wei, Mei Kang, Yin Tong, Zheng Wei, Peng Liu, Liping Wan, Xianmin Song

**Affiliations:** 1 Department of Hematology, Shanghai General Hospital, Shanghai Jiao Tong University, No. 100 Haining Road, 200080 Shanghai, China; 2 Clinical Research Center, Shanghai General Hospital, Shanghai Jiao Tong University, Shanghai, China; 3 Department of Hematology, Zhongshan Hospital, Fudan University, Shanghai, China

**Keywords:** Disease prevention, Acute myeloid leukaemia

## To the Editor:

For successful transplantation using a haploidentical donor, it requires effective depletion of T cells to reduce the risk of graft-versus-host disease (GvHD) and prevent serious GvHD. Contemporary practice of haploidentical hematopoietic stem cell transplantation (Haplo-HSCT) frequently adopts in vivo T-cell depletion strategies: a monoclonal antibody against T cells using antihuman thymocyte immunoglobulin (ATG) [1, 2] or posttransplant cyclophosphamide (PTCy) based regimen [3, 4].

The ATG-based regimen was one of the most commonly used GvHD prophylaxis for Haplo-HSCT in China but remaining an issue of higher risk of acute GvHD (aGvHD) up to 40% incidence [1, 2] as well as cytomegalovirus (CMV) and Epstein–Barr virus (EBV) infection. PTCy-based regimen had outstanding outcomes of GvHD prevention in Haplo-HSCT with bone marrow grafts with 21–34% of incidences of grades II–IV aGvHD. However, by substituting BM graft with peripheral blood stem cell (PBSC) grafts, the incidence of grades II–IV aGvHD increased in Haplo-HSCT even after the use of PTCy-based regimens for GvHD prophylaxis [5, 6]. Accordingly, there is room for further improvement of GvHD reduction when the haploidentical transplant was performed using a PBSC graft. Thus there is a practical demand for more powerful GvHD prophylaxis for haploidentical peripheral blood stem cell transplantation (Haplo-PBSCT) beyond ATG-based or PTCy-based regimens.

Recently, we have applied a novel regimen of combining low dose ATG (5 mg/kg) with low dose PTCy (one dose of 50 mg/kg) (low dose ATG/PTCy) for prevention of GvHD in Haplo-HSCT with PBSC grafts combined with unrelated single cord blood (UCB) in our center [7]. The novel GvHD prophylaxis regimen showed promising activity with grades II–IV aGvHD of 19.4%, which was significantly lower than that with other regimens including standard PTCy regimen (2 days Cy 100 mg/kg) [4–7]. Some studies [8–11] reported that UCB in combination with CD34^+^ selected PBSCs from a related mismatched donor were transplanted into recipients with fast engraftment, low incidences of GvHD, and promising long-term results. According to the results in these studies, an UCB was co-transfused with PBSCs in our study, while an UCB as the third party cells might obscure the effects of the novel regimen in the prophylaxis of GvHD. Thus to confirm the effects of the novel regimen on GvHD prophylaxis, two cohorts of patients received the same grafts (PBSCs plus a UCB) with standard-dose ATG or low doseATG/PTCy-based regimens were comparatively analyzed.

From January 2014 to June 2018, 36 patients with myeloid malignancies in standard-dose ATG group were enrolled in this trial, while 31 cases in low dose ATG/PTCy group were from June 2017. Eight cases in our previous study [7] were excluded from the current study because of five cases with ALL and three myeloid malignancy cases with loss to follow-up. Informed consent was obtained from all subjects, and the study protocol was approved by the local Ethical Review Board of the Shanghai General Hospital, Shanghai, China. The patients younger than 55 years old received myeloablative conditioning (MAC), while those equal to or older than 55 years of age received reduced-intensity conditioning (RIC). All patients received BFA conditioning regimens consisted of Fludarabine and Cytarabine with different doses of Busulfan (3.2 mg/kg/day for 3–4 days in cases of MAC,while 3.2 mg/kg/day for 2 days in RIC) [7, 12].

In the group received low dose ATG/PTCy, ATG was given at 2.5 mg/kg/day for 2 days (d − 2, d − 1; a total of 5 mg/kg) and a dose of cyclophosphamide (50 mg/kg/day) was given for 1 day on d + 3. Cyclosporine A (CSA) was given from d + 4. Mycophenolate mofetil (MMF) was administered orally at a dose of 1 g three times a day from d + 1 to d + 34 if aGvHD does not occur [7]. In the group received a standard-dose ATG-based regimen, ATG was given at a dose of 2.5 mg/kg/d from d − 4 to d − 1, a total of 10 mg/kg. CSA was given from d − 5. MMF was administered at a dose of 1 g three times a day from d + 1 to d + 30. Methotrexate (MTX) was added at a dose of 10 mg/m^2^ on d + 1, and 5 mg/m^2^ on days +3 and +6.

A total of 31 cases received a low dose ATG/PTCy, while 36 cases received a standard-dose ATG. No significant differences were observed in age, gender, HCT-CI, conditioning regimen, donor type, infused cell numbers of PBSCs, and UCB grafts between the two groups (Supplementary Table 1). The percentage of AML-CR1 patients in low dose ATG/PTCy group was significantly higher than that in standard-dose ATG group (32.3 vs. 8.3%, *P* = 0.014). While the percentage of MDS-MLD patients in low dose ATG/PTCy group was significantly lower than that in standard-dose ATG group (0.0 vs. 19.4%, *P* = 0.028). In addition, there was no significant difference (*P* = 0.422) between two groups in revised-disease risk index (R-DRI). As expected, the follow-up duration was longer in standard ATG group (median 23 months) than in low dose ATG/PTCy group (median 12.5 months; *P* = 0.004) because the use of low dose ATG/PTCy had been recently introduced while the standard-dose ATG regimen was a standard GvHD prophylaxis for longer period of time in our center.

With respect to engraftment, no differences were noted between the two groups in neutrophil (*P* = 0.712) and platelet engraftment (*P* = 0.312). The primary graft failure rate was similar: 3.2% (*n* = 1/31) in the low dose ATG/PTCy group and 2.8% (*n* = 1/36) in the standard-dose ATG group (*P* = 1.000). Secondary graft failures (SGFs) were observed in two cases without donor chimerism (graft rejection) in low dose ATG/PTCy group, which occurred at +24 d and +80 d after transplantation in the context of infection. The two cases were diagnosed with AML and received MAC regimens. Although no patient developed SGF in the standard-dose ATG group, there was no difference in the risk of SGF (*P* = 0.272) between two groups.

In terms of GvHD risk, the risk of aGvHD was significantly lower in the low dose ATG/PTCy group: the incidence of grades II–IV aGvHD was 17.0% in low dose ATG/PTCy (*n* = 5) within 180 days vs. 40.2% in standard ATG group (*n* = 14; *P* = 0.042; Table 1 and Supplementary Fig. 1).There was no significant differences in the incidence of chronic GVHD between the two groups. While, the rate of moderate to severe cGvHD was significantly lower as 11.2% at 1-year in low dose ATG/PTCy group as compared to 40.1% in the standard-dose ATG group (*P* = 0.029). The low dose ATG/PTCy group seemed to have a reduced trend of CMV and EBV infection compared to the standard-dose ATG group (Table 1).

**Table 1:** Clinical outcomes of patients in the two groups.

	Low dose ATG/PTCy regimen (*n* = 31)	Standard-dose ATG regimen (*n* = 36)	*P*
CI of grades I–IV aGvHD at 180 days	49.7%	79.3%	0.065
CI of grades II–IV aGvHD at 180 days	17.0%	40.2%	0.042
CI of grades III–IV aGvHD at 180 days	3.2%	23.1%	0.025
CI of cGVHD at 1 year	42.7%	57.7%	0.190
CI of moderate to severe cGVHD at 1 year	11.2%	40.1%	0.029
Viral infection			
CMV viremia	13 (41.9%)	23 (63.8%)	0.072
CMV disease	5 (16.1%)	5 (13.9%)	1.000
EB viremia	14 (45.2%)	24 (66.7%)	0.076
PTLD EBV related	0 (0.0%)	1 (2.8%)	1.000
CIR at 1 year	22.9%	8.7%	0.042
NRM at 1 year	12.9%	36.2%	0.038
Causes of NRM			
GvHD	0 (0%)	7 (23.3%)	0.028
Infection	0 (0%)	5 (13.9%)	0.091
MOF	1 (3.2%)	1 (2.8%)	1.000
Graft failure/dysfunction	3 (9.7%)	1 (2.8%)	0.502
Early mortality (Within 3 months post transplant)	3 (9.7%)	11 (30.6%)	0.036
OS (1 year)	74.9%	59.4%	0.255
LFS (1 year)	64.2%	55.0%	0.400

*CMV* cytomegalovirus, *PTLD* posttransplant lymphoproliferative disorder, *CIR* cumulative incidence of relapse, *CI* cumulative incidence, *NRM* non-relapse mortality, *MOF* multiple organ failure, *OS* overall survival, *LFS* leukemia-free survival.

However, long-term transplant outcomes did not show any significant differences between the two groups concerning overall survival (*P* = 0.255) and leukemia-free survival (*P* = 0.400; Table 1 and Supplementary Fig. 2). The non-relapse mortality rate was lower in the low dose ATG/PTCy group as 12.9% at 1 year than in standard-dose ATG group (36.2%; *P* = 0.038), while the incidence of relapse was higher in the low dose ATG/PTCy group as 22.9% at 1 year than in the standard-dose ATG group (8.7%; *P* = 0.042).

The key finding of the present study is that the risk of grades II–IV aGvHD and moderate to severe cGvHD were significantly lower in the low dose ATG/PTCy group than that in the standard ATG group, suggesting that low dose ATG/PTCy regimen is very promising for prevention of GvHD in Haplo-PBSCT which could potentially improve GvHD-free, relapse-free survival after Haplo-HSCT. The rationale of using a combined regimen of ATG and PTCy is that administration of low dose ATG could deplete early active T-lymphocytes while an infusion of PTCy on day +3 could eradicate rapidly proliferating T cells after antigen exposure [4], which could exert a synergistic activity to reduce the risk of GvHD because they have different action mechanisms on T-lymphocyte depletion.

Of note, the low dose ATG/PTCy regimen can reduce the risk of NRM significantly: 12.9% of NRM in the low dose ATG/PTCy group vs. 36.2% of NRM in the standard ATG group. It can be explained by the extremely lower rate of GvHD related death in the low dose ATG/PTCy group compared to that in the standard-dose ATG group (*P* = 0.028). The incidences of infection were not significantly different between the two groups. However, the infection-related mortality rate was substantially higher in the standard ATG group compared to that in the low dose ATG/PTCy group (9/36 vs. 1/31, *P* = 0.032). Our personal experience is that the infectious complications in the cases received standard-dose ATG were much more challenging to control than those received low dose ATG/PTCy. It could be plausible that standard-dose ATG is more immunosuppressive, especially for T-cell immunity than low dose ATG/PTCy regimen, thus resulting in prolonged immunosuppression.

The risk of relapse was higher in the low dose ATG/PTCy group compared to the standard ATG group (22.8 vs. 8.7%). Of note, more patients died at earlier time (within 3 months) in the standard-dose ATG group (30.6%) compared to the low dose ATG/PTCy group (9.7%, *P* = 0.036), which might be the major reason for a lower relapse rate in the standard-dose ATG group because the median relapse time was 7 months in overall patients. The relapse incidences in the present study were higher than that in Ruggeri’s et al. [13] and Salvatore’s et al. [14] studies. However, it requires cautious interpretation because of different disease characteristics among the studies. Our study included more patients with advanced disease close to 60% (19/31 and 21/36, respectively), while higher numbers of patients in remission were included in the Ruggeri’s et al. [13] (100% in CR1) and Salvatore’s et al. [14] studies (69% in CR1).

Although it did not reach statistical significances, there were strong trends of reduced incidences of CMV and EBV infection in the low dose ATG/PTCy group in comparison to the standard-dose ATG group. The incidence of CMV viremia in the low dose ATG/PTCy group was similar to that with standard PTCy regimen in a range of 38–50% [4, 15, 16] of CMV viremia, while it was much higher when received ATG-based regimens with the incidence of CMV viremia over 60% [2].

In summary, the low dose ATG/ PTCy was feasible and could effectively reduce the risk of GvHD and lower the NRM in comparison to the standard-dose ATG. A prospective randomized trial would be required to reach a definite conclusion on the superior efficacy of low dose ATG/PTCy regimen in Haplo-PBSCT.

## Supplementary information

Supplementary Table1

supplementary FIGURE LEGENDS

figure s1

figure s2
