# Supplementary material for: Low dose anti-thymocyte globulin with low dose posttransplant cyclophosphamide (low dose ATG/PTCy) can reduce the risk of graft-versus-host disease as compared with standard-dose anti-thymocyte globulin in haploidentical peripheral hematopoietic stem cell transplantation combined with unrelated cord blood
Source: Bone Marrow Transplant. 2020 Sep 1;56(3):705–8. doi: 10.1038/s41409-020-01047-2 (PMC7943423; doi:10.1038/s41409-020-01047-2)
Supplement: Supplementary file 1 — Supplementary Table1 [file 41409_2020_1047_MOESM1_ESM.docx]

Supplementary Table1.The characteristics of disease, patients and donors between the two groups

|  | Lowdose ATG/PTCy | Standard dose ATG | *P* |
| --- | --- | --- | --- |
| No. | 31 | 36 | - |
| Median follow-up(m, range) | 12.5(6-18.5) | 23(6-59) | 0.004 |
| Median age (y, range) | 43(8-68) | 37(15-67) | 0.253 |
| Gender (male/female) | 17/14 | 24/12 | 0.322 |
| Disease status  AML-CR1  AML≥CR2  Refractory/relapse AML  MDS-MLD  MDS-EB1  MDS-EB2  CMML  CML in accelerated phase | 10(32.3%)  2(6.5%)  11(35.5%)  0(0.0%)  1(3.2%)  4(12.9%)  3(9.7%)  0(0.0%) | 3(8.3%)  2(5.6%)  15(41.7%)  7(19.4%)  3(8.3%)  5(13.9%)  0(0.0%)  1(2.8%) | 0.004  0.014  1.000  0.605  0.028  0.717  1.000  0.208  1.000 |
| Blasts in bone marrow prior to HSCT (≥25%) | 7(22.6%) | 6(16.7%) | 0.542 |
| R-DRI  Low  Intermediate  High  Very high | 0(0.0%)  15(48.4%)  16(51.6%)  0(0.0%) | 0(0.0%)  14(38.9%)  21(58.3%)  1(2.8%) | 0.422 |
| HCT-CI score  0  1  2  ≥3 | 25(80.6%)  5(16.1%)  0(0.0%)  1(3.2%) | 28(77.8%)  6(16.7%)  1(2.8%)  1(2.8%) | 0.827 |
| Conditioning regimen  RIC  MAC | 5(16.1%)  26(83.9%) | 6(16.7%)  30(83.3%) | 0.953 |
| Donors  Father  Mother  Sibling  Offspring  Cousin | 6(19.4%)  1(3.2%)  5(16.1%)  16(51.6%)  3(9.7%) | 9(25.0%)  3(8.3%)  8(22.2%)  15(41.7%)  1(2.8%) | 0.563 |
| Donors  Gender (male/female)  Age(≤30 / >30y)  Blood type of donor and recipient (matched/unmatched)  Gender between donor and recipient  Female to male  Female to female  Male to female  Male to male | 20/11  14/17  17/14  5(16.1%)  6(19.4%)  8(25.8%)  12(38.7%) | 21/15  16/20  17/19  11(30.6%)  4(11.1%)  13(36.1%)  8(22.2%) | 0.605  0.953  0.534  0.227 |
| PBSC graft  MNC (×10^8^/kg)  CD34^+^ cells (×10^6^/kg)  CD3^+^ cells (×10^8^/kg) | 14.4(5.5-26.3)  13.1(2.4-31.7)  3.4(1.9-10.8) | 15.3(6.5-21.7)  10.7(4.5-29.3 )  3.6(2.3-8.8) | 0.211  0.597  0.262 |
| UCB  Nucleated cells(×10^7^/kg)  CD34+ cells (×10^4^/kg) | 1.9(1.4-5.6)  4.9(1.5-11.2) | 2.1(1.1-6.8)  5.1(1.8-13.6) | 0.354  0.763 |

AML: acute myeloid leukemia; CR: complete remission; MDS: myelodysplastic syndrome;MLD: multilineagedysplasia; EB1: excess blasts-1;EB2: excess blasts-2;CMML:chronic myelomonocytic leukemia; CML: chronic myelogenous leukemia; R-DRI: refined Disease Risk Index; HCT-CI: Hematopoietic Cell Transplant-Co-morbidity Index; RIC: reduced-intensity conditioning; MAC: myeloablative conditioning; MNC: mononuclear cells.
