# Supplementary material for: Low dose anti-thymocyte globulin with low dose posttransplant cyclophosphamide (low dose ATG/PTCy) can reduce the risk of graft-versus-host disease as compared with standard-dose anti-thymocyte globulin in haploidentical peripheral hematopoietic stem cell transplantation combined with unrelated cord blood
Source: Bone Marrow Transplant. 2020 Sep 1;56(3):705–8. doi: 10.1038/s41409-020-01047-2 (PMC7943423; doi:10.1038/s41409-020-01047-2)
Supplement: Supplementary file 2 — supplementary FIGURE LEGENDS [file 41409_2020_1047_MOESM2_ESM.pdf]

FIGURE LEGENDS

SupplementaryFigure 1. The cumulative incidences (CIs) of GvHD after Haplo-HSCT. The CIs of grades II-IV aGvHD, grades III-IV aGvHD, cGvHD and moderate to severe cGvHD were shown in (A), (B), (C) and (D),respectively. The CIs of grades II-IV aGvHD and grades III-IV aGvHD were significantly lower in the low dose ATG/PTCy than that in the standard ATG dose group (*P*=0.042 and *P*=0.025,respectively).There was no significant difference concerning the CIs of cGvHD (*P*=0.190) between the two groups. The CI of moderate to severe cGvHD was significantly lower in the low dose ATG/PTCy than that in the standard ATG dose group(*P*=0.029).

SupplementaryFigure 2. The cumulative incidences(CIs) of relapse and NRM (A), the overall survival (OS) (B), and the leukemia free survival (LFS) (C).The CI of relapse was significantly higher in the low dose ATG/PTCy than that in the standard ATG dose group (*P*=0.042) . The CI of NRM was significantly lower in the low dose ATG/PTCy than that in the standard ATG dose group(*P*=0.038). There were no significant differences concerning OS (*P*=0.255), and LFS(*P*=0.400)between the two groups.
