# Supplementary figures and images for: Low dose anti-thymocyte globulin with low dose posttransplant cyclophosphamide (low dose ATG/PTCy) can reduce the risk of graft-versus-host disease as compared with standard-dose anti-thymocyte globulin in haploidentical peripheral hematopoietic stem cell transplantation combined with unrelated cord blood
Source: Bone Marrow Transplant. 2020 Sep 1;56(3):705–8. doi: 10.1038/s41409-020-01047-2 (PMC7943423; doi:10.1038/s41409-020-01047-2)

Supplementary Figure 1.

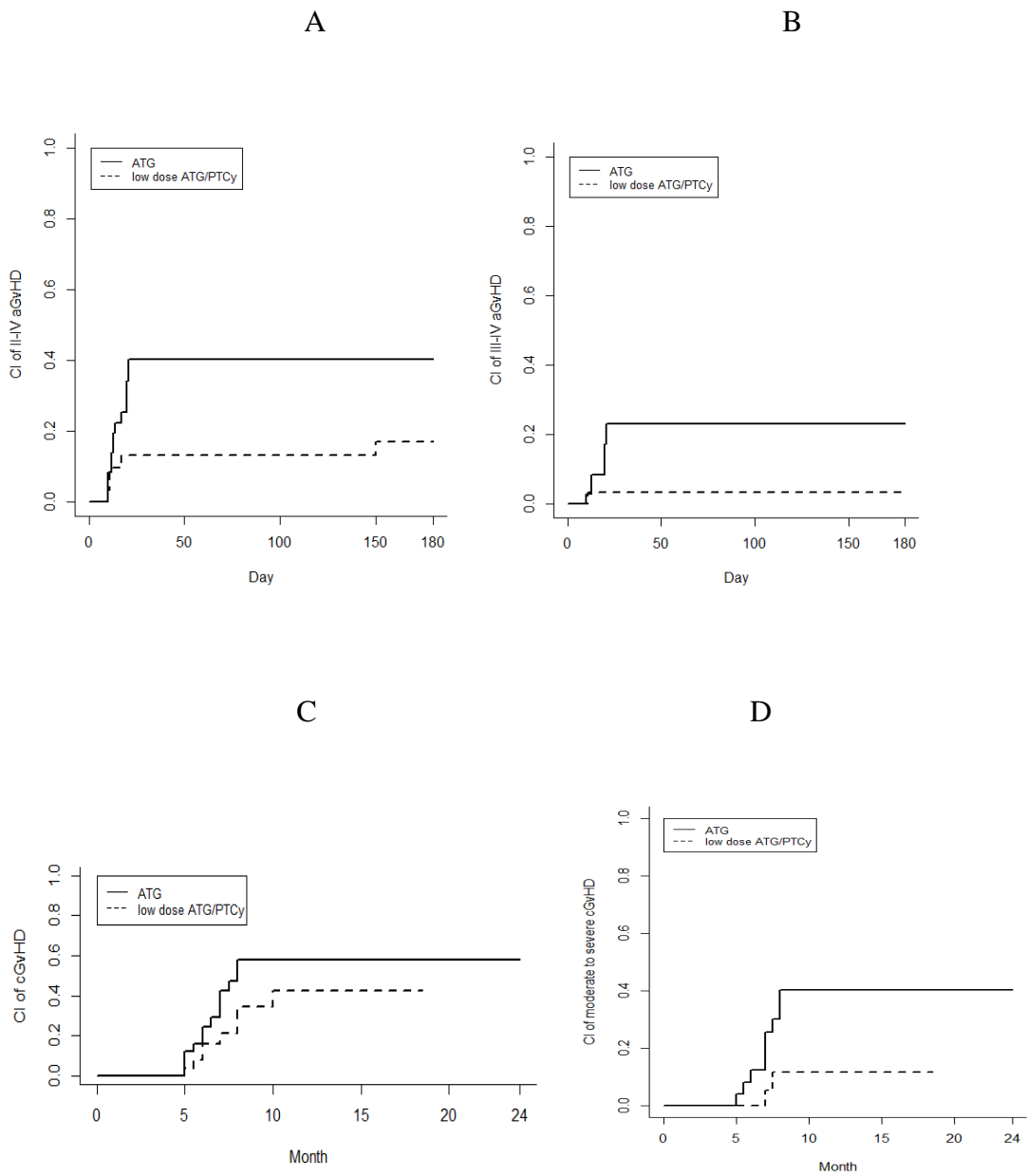

Supplement: Supplementary file 3 — figure s1 [file 41409_2020_1047_MOESM3_ESM.pdf]

Supplementary Figure 2.

A

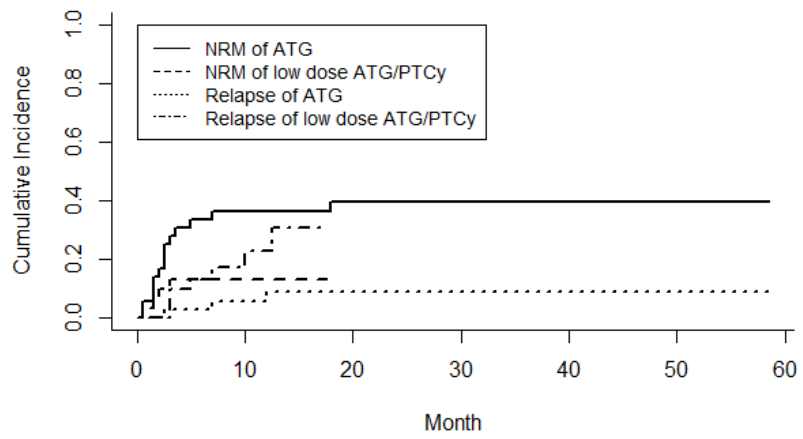

B

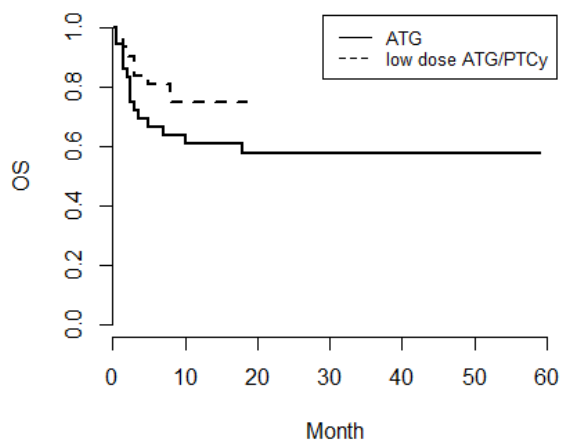

C

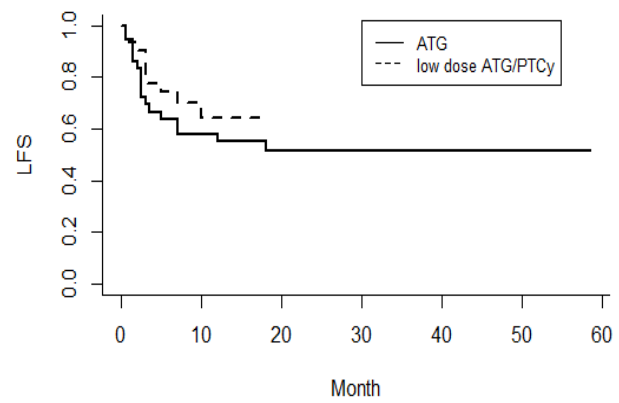

Supplement: Supplementary file 4 — figure s2 [file 41409_2020_1047_MOESM4_ESM.pdf]
